# Glutathione Transferase from *Trichoderma virens* Enhances Cadmium Tolerance without Enhancing Its Accumulation in Transgenic *Nicotiana tabacum*

**DOI:** 10.1371/journal.pone.0016360

**Published:** 2011-01-21

**Authors:** Prachy Dixit, Prasun K. Mukherjee, V. Ramachandran, Susan Eapen

**Affiliations:** Nuclear Agriculture and Biotechnology Division, Bhabha Atomic Research Centre, Mumbai, India; Purdue University, United States of America

## Abstract

**Background:**

Cadmium (Cd) is a major heavy metal pollutant which is highly toxic to plants and animals. Vast agricultural areas worldwide are contaminated with Cd. Plants take up Cd and through the food chain it reaches humans and causes toxicity. It is ideal to develop plants tolerant to Cd, without enhanced accumulation in the edible parts for human consumption. Glutathione transferases (GST) are a family of multifunctional enzymes known to have important roles in combating oxidative stresses induced by various heavy metals including Cd. Some GSTs are also known to function as glutathione peroxidases. Overexpression/heterologous expression of GSTs is expected to result in plants tolerant to heavy metals such as Cd.

**Results:**

Here, we report cloning of a glutathione transferase gene from *Trichoderma virens*, a biocontrol fungus and introducing it into *Nicotiana tabacum* plants by *Agrobacterium-*mediated gene transfer. Transgenic nature of the plants was confirmed by Southern blot hybridization and expression by reverse transcription PCR. Transgene (*TvGST*) showed single gene Mendelian inheritance. When transgenic plants expressing *TvGST* gene were exposed to different concentrations of Cd, they were found to be more tolerant compared to wild type plants, with transgenic plants showing lower levels of lipid peroxidation. Levels of different antioxidant enzymes such as glutathione transferase, superoxide dismutase, ascorbate peroxidase, guiacol peroxidase and catalase showed enhanced levels in transgenic plants expressing *TvGST* compared to control plants, when exposed to Cd. Cadmium accumulation in the plant biomass in transgenic plants were similar or lower than wild-type plants.

**Conclusion:**

The results of the present study suggest that transgenic tobacco plants expressing a *Trichoderma virens* GST are more tolerant to Cd, without enhancing its accumulation in the plant biomass. It should be possible to extend the present results to crop plants for developing Cd tolerance and in limiting Cd availability in the food chain.

## Introduction

Increased industrialization, urbanization and anthropogenic activities have enhanced the levels of metal pollutants in the environment, which ultimately enter the food chain causing damage to life forms. Cadmium (Cd) is a widespread toxic heavy metal released into the environment by traffic, power substations, cement factories, metal industries, rock mineralization and by the use of superphosphate fertilizers [1]. Cadmium taken up by plants enters the food chain and causes toxicity to animals and humans who consume it (http://www.accessscience.com/studycenter.aspx). Exposure to low levels of Cd is associated with renal dysfunction, pulmonary emphysema and bone demineralization. High concentrations of Cd have been found to be carcinogenic, mutagenic and teratogenic to a large number of animal species including humans [2]. Agricultural fields worldwide are contaminated with Cd especially due to the increased use of superphosphate fertilizers and plants take up Cd and accumulate it in the edible parts. High levels of Cd are toxic and limits yield of plants. Attempts are therefore being made to develop plants which are tolerant to Cd, without altering and preferably lowering its accumulation. In addition to the conventional methods of plant improvement, development of transgenic plants for Cd tolerance is an upcoming area of research.

Glutathione transferases (GSTs) (E.C. 2.5.1.18) are a family of multifunctional enzymes which are known to play important roles in combating different biotic and abiotic stresses including heavy metal stress through amelioration of oxidative damage [3]. Many biotic and abiotic stresses are known to lead to increased production of reactive oxygen species causing oxidative damage to DNA and lipids in plants. Glutathione transferases can eliminate membrane lipid peroxides such as 4-hydroxyalkenals as well as products of oxidative DNA degradation such as base propanol by conjugating them with glutathione (GSH) [3], [4]. Many glutathione transferases also have glutathione peroxidase activity [5] and they detoxify lipid peroxides directly [3], [6].

Different abiotic stresses including exposure to heavy metals like Cd is known to induce increased production of reactive oxygen species (ROS) such as singlet oxygen, superoxide anion, hydrogen peroxide and hydroxyl radicals [7], [8], [9] which lead to membrane lipid peroxidation resulting in production of cytotoxic 4-hydroxy alkenals [10]. These reactive electrophiles inhibit DNA and protein synthesis [10]. Other consequence of ROS production includes formation of base propanols, which are highly cytotoxic products of oxidative DNA damage [11]. The excess ROS are scavenged by both enzymatic and non-enzymatic mechanisms [12], [13] in plant cells in order to protect them from ROS induced damage. Plants have an array of enzymes such as glutathione transferase (GST), superoxide dismutase (SOD), ascorbate peroxidase (APX), guiacol peroxidase (GPX) and catalase (CAT), which scavenge the different ROS, thus protecting the cells and organelles from tissue disfunction [14]. Superoxide dismutase (SOD) is a key enzyme, which dismutates superoxide free radical (O_2_^−^) to H_2_O_2_ and oxygen. Since H_2_O_2_ is toxic to cells, catalase (CAT) and peroxidase detoxify it to water and oxygen. Glutathione transferases, which also function as glutathione peroxidase are known to catalyze the reduction of organic hydroperoxides to less toxic monohydroxy alcohols [15]. Glutathione transferases are also known to be involved in glutathione-Cd formation, decreasing Cd stress in yeast cells [16].

In plants and animals, GST expression has been closely associated with stress [11]. Studies by Moons [17] showed that different metals including Cd enhanced the expression of GST genes such as *Osgstu*3 and *Osgstu*4 in rice plants, indicating that GST expression is associated with Cd stresses. The increased GST levels appear to protect organisms from oxidative stress. Overexpression of tobacco GST was shown to enhance the growth of transgenic tobacco seedlings grown under different stresses [18]. Studies in rice transgenics overexpressing GST and catalase showed enhanced tolerance to Cd [19]. Hence overexpression/ heterologous expression of GST should result in transgenic plants tolerant to heavy metals such as Cd. Since Cd causes toxicity in humans, it is desirable that Cd tolerant plants should have reduced or similar Cd content in the biomass/ edible parts. Although attempts have been made in the past to develop Cd tolerant plants by incorporation of heterologous genes [20]–[22], it also resulted in enhanced Cd accumulation. Increased Cd acquisition is useful for phytoremediation; however, for crop plants, increased Cd acquisition is an undesirable trait. Since our earlier work has shown that genes from *Trichoderma virens* express well in plants [23], in the present study we selected a unique and highly expressed GST gene from *T. virens* for cloning and introduction into plants.

Here we report the development of transgenic tobacco plants expressing a GST gene from *Trichoderma virens* and testing their tolerance to Cd and also acquisition of Cd. Since Cd induced oxidative stress is known to lead to free radical mediated lipid peroxidation, the levels of lipid peroxidation in transgenic plants exposed to Cd was assessed. To understand the biochemical detoxification mechanism that the transgenic plant with a fungal GST gene has developed to combat oxidative stress induced by Cd, different antioxidant enzymes such as GST, SOD, APX, GPX and CAT were monitored. Our data suggest that *TvGST* gene could enhance the tolerance of *N. tabacum* plants to Cd, without enhancing its accumulation.

## Results

### Cloning of GST gene from *Trichoderma virens*

The *T. virens* GST (herein designated as *TvGST*) ORF is 1008 bp long and is interrupted by four introns of 61, 57, 54 and 77 bp respectively. The predicted protein is 252 amino acid long and possesses an N-terminal thioredoxin-fold domain, called the prion domain of Ure2p and a C-terminal alpha helical domain. In addition to its role in nitrogen regulation, Ure2p is also known to confer protection to cells against heavy metal ion and oxidant toxicity and shows both glutathione transferase and glutathione peroxidase activities (http://www.ncbi.nlm.nih.gov/Structure/cdd/cdd.shtml). It is highly homologous to GSTs from other Ascomycetous fungi (Figure S1). The gene was cloned into the pTZ57RT vector and later introduced into plant expression vector.

### Development of transgenic plants expressing GST

**Figure 1:**
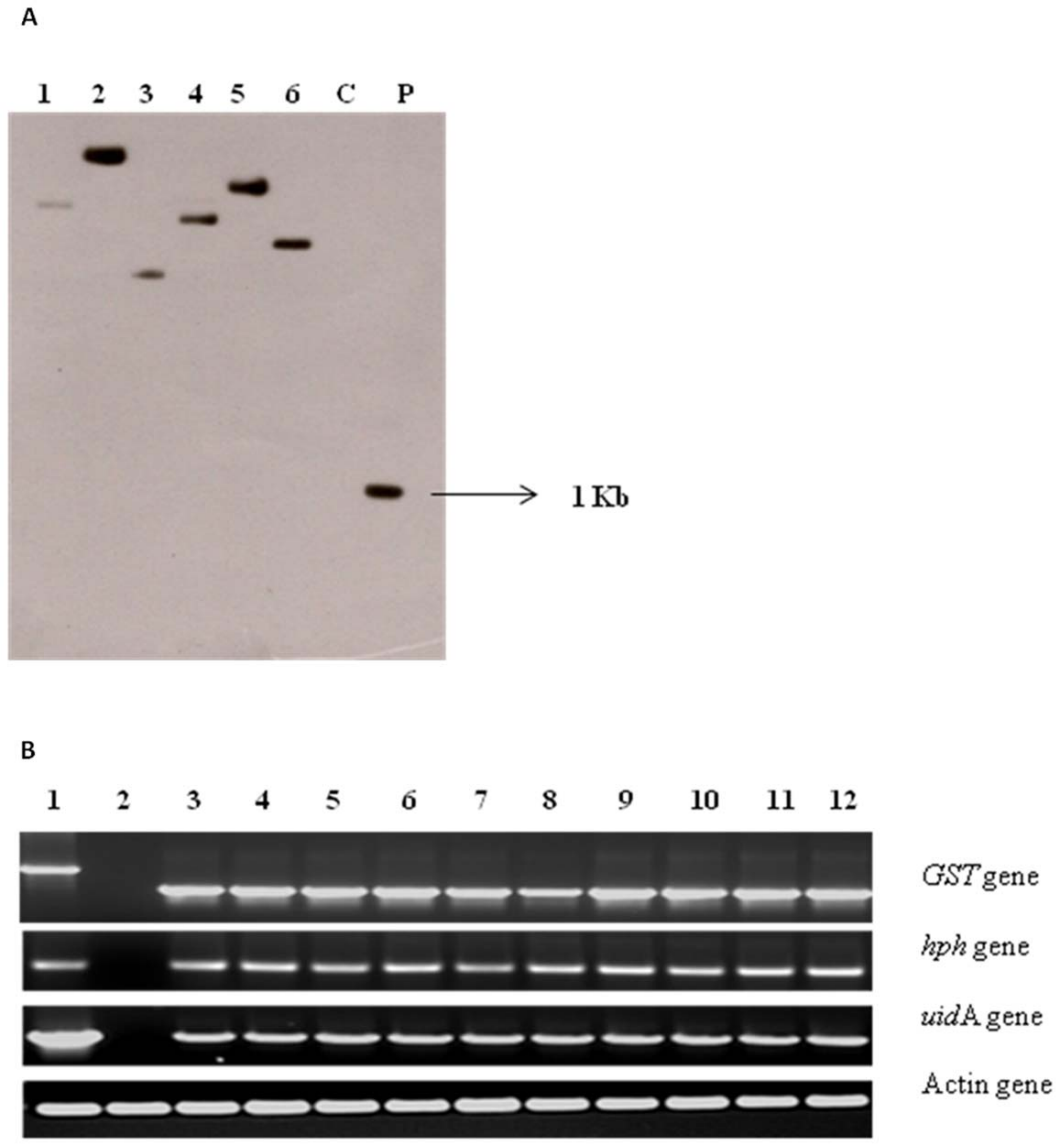
A Southern blot hybridization of genomic DNA of tobacco plants hybridized with GST probe. C- non-transgenic plant, P-*ECoR*I- *HindIII* digested 1 Kb fragment of GST-pCAMBIA 1301 plasmid as positive control,1–6 transgenic plants showing the bands of different sizes, indicating the integration of *GST* gene at different loci in the genome of transgenic plants. **B** RT-PCR of PCR positive transgenic tobacco plants. 1-pCAMBIA 1301-GST plasmid, 2-non-transgenic plant, 3–12 PCR positive transgenic plant samples. All PCR positive transgenic lines were found positive in RT-PCR. Actin gene was used as the house- keeping gene.

Glutathione transferase gene from *Trichoderma virens* (*TvGST*) was cloned, introduced into tobacco and transgenic plants developed. Stable transformation frequency was 80% based on the number of explants showing regeneration on MS medium [24] supplemented with hygromycin B. Tobacco leaf disc explants after 48 hours of co-culture showed transient expression of *uid*A gene (Figure S2). Stable expression of *uid*A gene was seen 5–6 weeks after transformation (Figure S2). The putative transgenic plants showed amplification of expected band size of 1 Kb for *TvGST* gene, while control plants did not show any amplification when subjected to PCR amplification with specific primers (Figure S3). Southern blot hybridization with genomic DNA from transgenic plants showed a single fragment greater than 1 Kb, while the control plant did not show any signal (Figure 1A). Results of RT-PCR using total RNA from putative transgenic plants confirmed that *TvGST* gene got transcribed in all selected transgenic lines, while there was no transcription in non-transgenic control lines (Figure 1B).

### GST activity assay of T_0_ *TvGST* expressing plants

**Figure 2:**
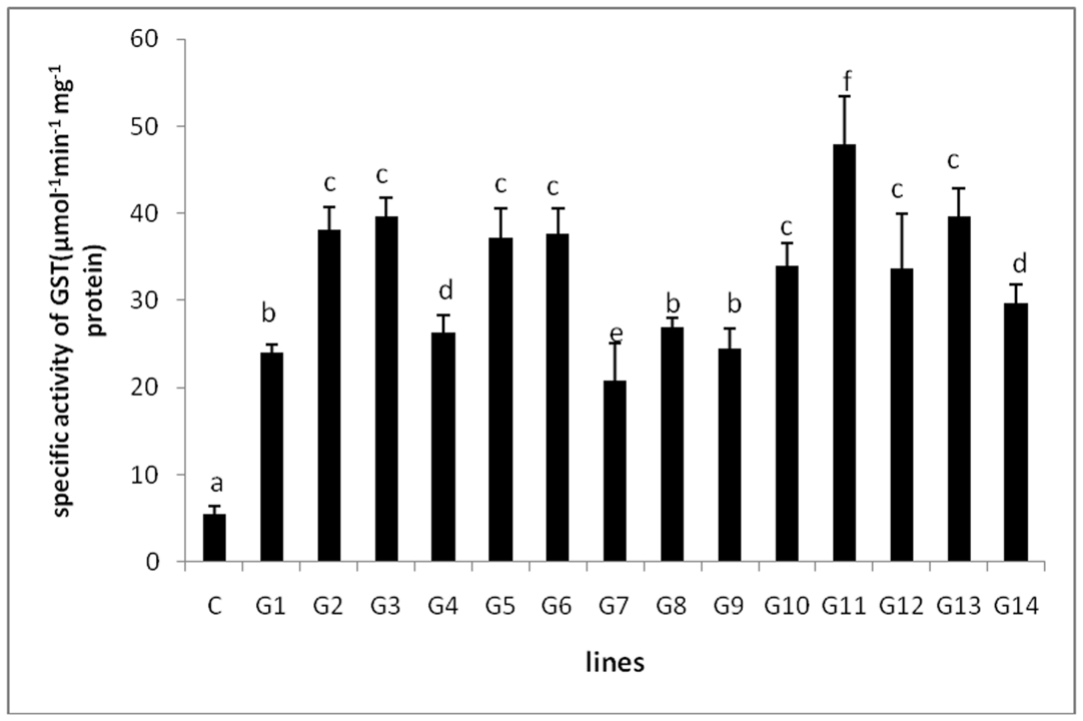
Enzymatic assay for GST using transgenic and control tobacco plants. C-control plant, G1-G14 transgenic plants showing different levels of expression of GST enzyme. G11 transgenic line showed the maximum (∼6 fold) expression of GST as compared to control plants. Different letters indicate significantly different values at *p*≤0.05 by Duncan's test.

Fourteen transgenic plants (T_0_) along with controls when tested, GST activity in the leaves of all tested transgenic plants was found to be significantly higher as compared to control tobacco plants (Figure 2). Out of 14 transgenic plants tested for GST activity, six plants showed high levels of expression of GST (6–8 times) as compared to control and these plants were selected for further analysis. Among the different lines tested, plant G11 showed the highest GST activity.

### Segregation analysis of T_1_ generation

Segregation analysis in the T_1_ generation showed a Mendelian ratio of 3∶1 in all the tested lines (Table S2), indicating the integration of *hph* transgene at a single locus. T_1_ hygromycin resistant transgenic plants showed the presence of *TvGST* gene on PCR amplification, confirming the presence of this transgene in T_1_ plants.

### Cadmium tolerance studies

**Figure 3:**
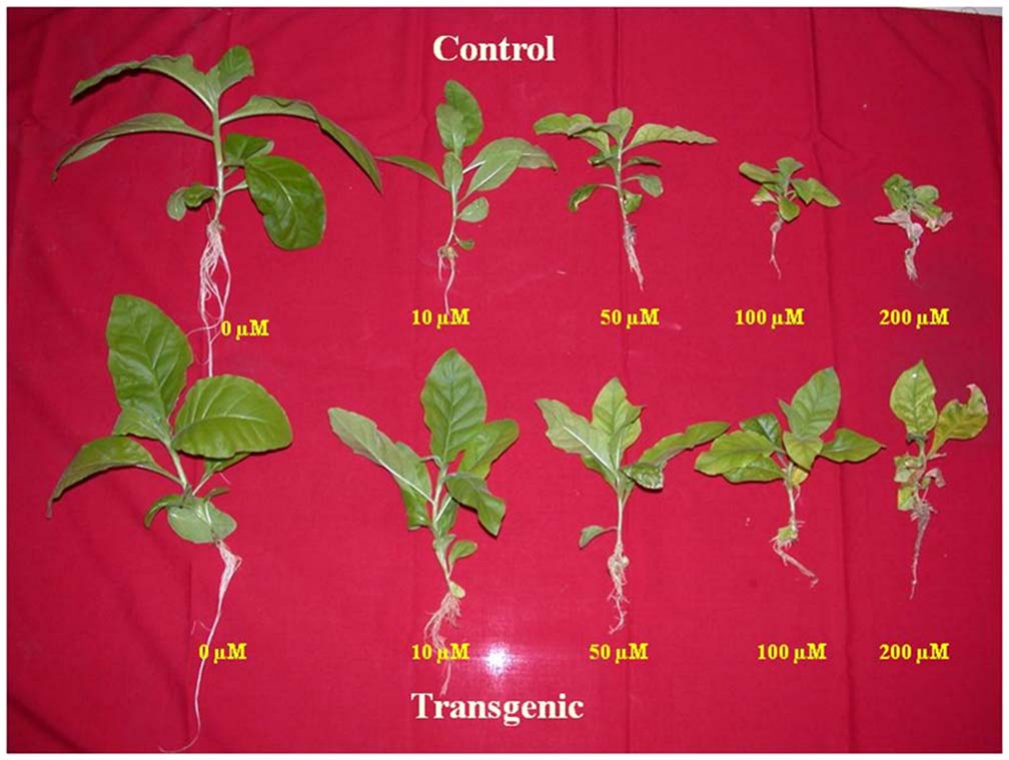
Control and transgenic plants grown in varying Cd concentrations.

When transgenic tobacco plants (T_0_, G11 line) expressing *TvGST* gene from *Trichoderma virens*, were exposed to different concentrations of Cd (10, 50, 100, 200 and 400 µM), they showed enhanced tolerance as compared to non-transgenic plants. Both control and transgenic plants could grow in the presence of Cd up to 50 µM concentration without any visible toxicity symptoms, but higher concentrations of Cd caused a reduction in growth and biomass of both transgenic and wild type plants (Figure 3). In general, transgenic plants expressing *TvGST* gene showed better growth and enhanced tolerance to Cd at 10, 50, 100 and 200 µM compared to control plants (Figure 3). Transgenic plants showed 45% reduction in biomass as compared to 71% in control plants when exposed to 100 µM Cd concentration (Figure S4). At 200 µM Cd concentration, growth of control plants was severely inhibited, with 84% reduction in biomass as compared to 56% in transgenic plants (Figure S4). Hence transgenic T_0_ plants showed better growth than wild-type plants when exposed to Cd upto 200 µM Both control and transgenic plants failed to survive when exposed to 400 µM Cd. In addition to T_0_ plants, five independent T_1_ transgenic plants grown *in vitro* showed enhanced tolerance and better growth than wild-type plants when exposed to Cd at 100 and 200 µM (Figure S5, S6).

### Antioxidant assay of plants exposed to Cd

**Figure 4:**
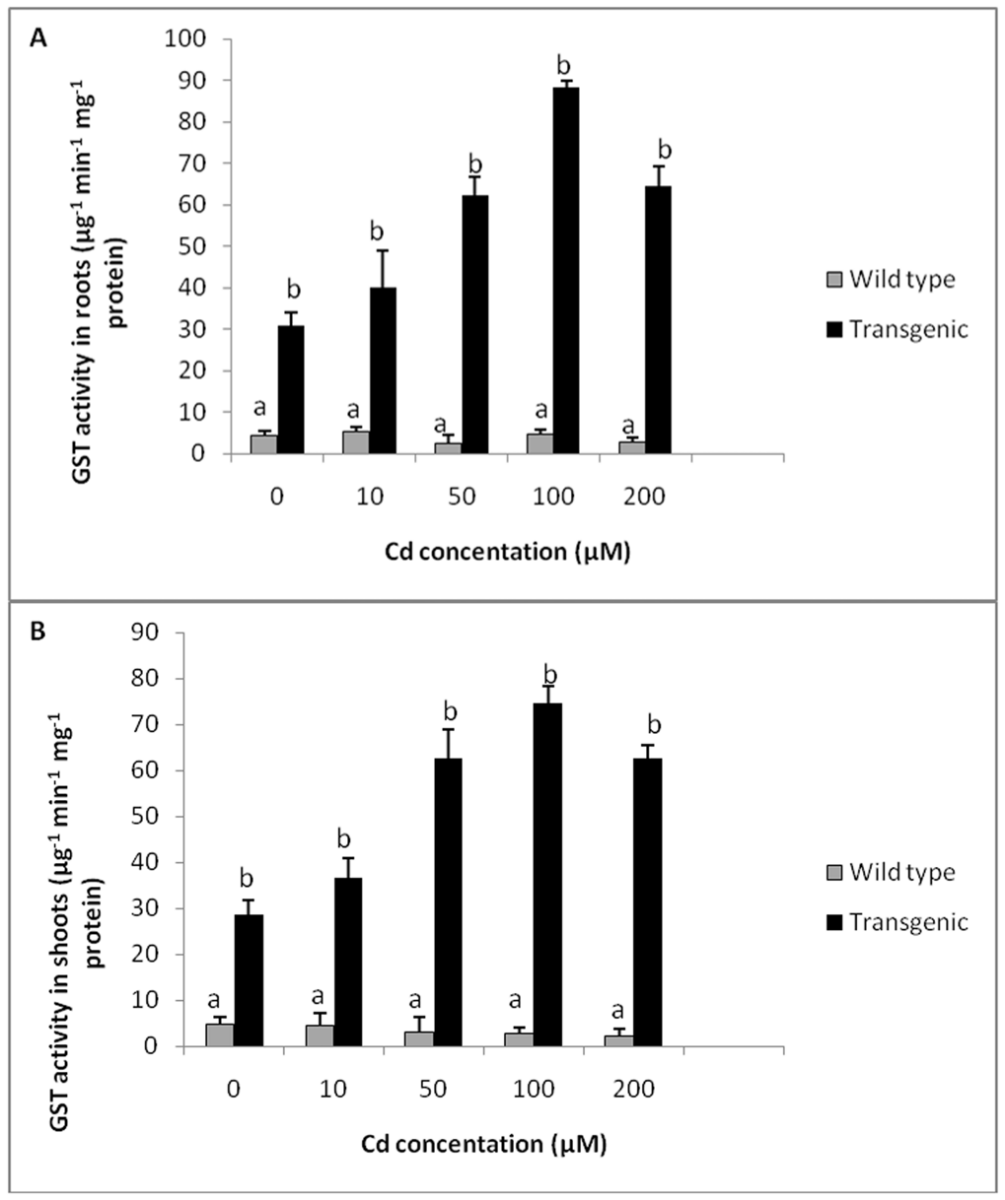
GST activity in transgenic and wild type plants exposed to different Cd concentrations. **A** GST activity in roots of transgenic and wild type plants exposed to different Cd concentrations. **B** GST activity in shoots of transgenic and wild type plants exposed to different Cd concentrations. All values are means of three replicates. Different letters indicate significantly different values at p≤0.05.

Levels of GST in roots and shoots of transgenic tobacco plants were found to be about 7 times higher as compared to wild type plants in the absence of any treatment. When medium was spiked with Cd, with an increase in Cd concentration, there was an increase in GST activity in both roots and shoots of transgenic tobacco plants up to 100 µM Cd (Figure 4A, B). Wild type plants did not show any significant change in GST activity at all Cd concentrations tested. Roots of transgenic plants showed a maximum of 19 times higher GST activity at 100 µM Cd as compared to wild type plants, whereas shoots showed a maximum of 27 times more GST activity compared to wild type plants at 100 µM Cd concentration. In general, glutathione transferase activity of transgenic plants although showed a decline at 200 µM Cd, was much higher than that of wild-type plants (Figure 4A, B). Glutathione transferase was significantly higher in transgenic plants as compared to control plants when exposed to different concentrations of Cd.

### Antioxidant enzyme assay

**Figure 5:**
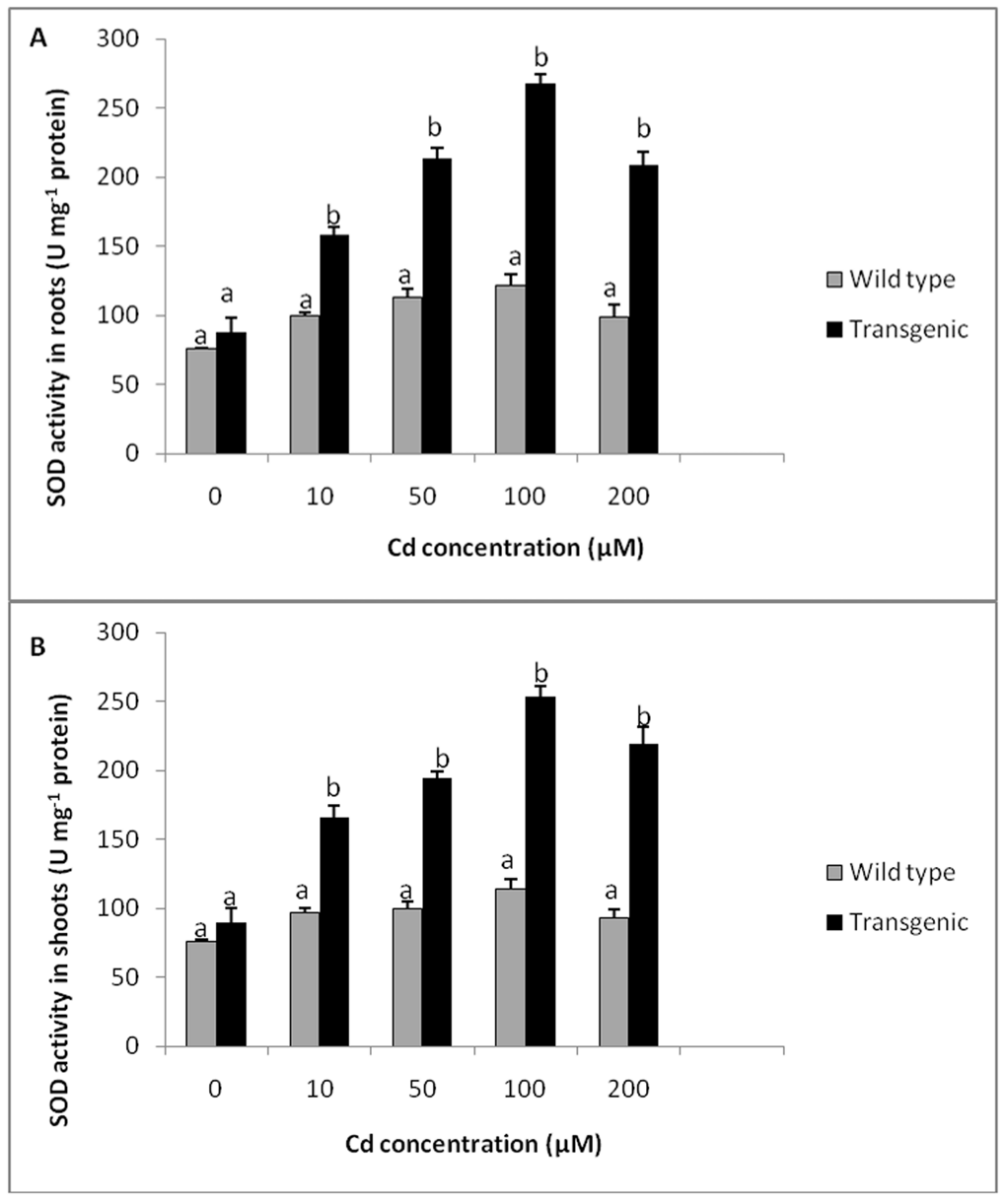
SOD activity in transgenic and wild type plants exposed to different Cd concentrations. **A** SOD activity in roots of transgenic and wild type plants exposed to different Cd concentrations. **B** SOD activity in shoots of transgenic and wild type plants exposed to different Cd concentrations. All values are means of three replicates. Different letters indicate significantly different values at p≤0.05.

Roots and shoots of wild type and transgenic plants did not show any significant differences in SOD activity when grown in medium without Cd. However, both transgenic as well as wild type plants showed an increase in SOD activity with increase in Cd concentrations up to 100 µM. The levels of SOD activity was more than two fold higher in transgenic plants as compared to wild type plants exposed to Cd at 100 µM. Further increase in Cd concentrations (200 µM) caused a decline in SOD activity in transgenic plants, although it was 2.1 times higher as compared to wild type plants in roots and 2.3 times higher in shoots. (Figure 5A, B). In general, SOD levels, in transgenic plants exposed to Cd (10, 50, 100 and 200 µM) were significantly higher compared to control.

**Figure 6:**
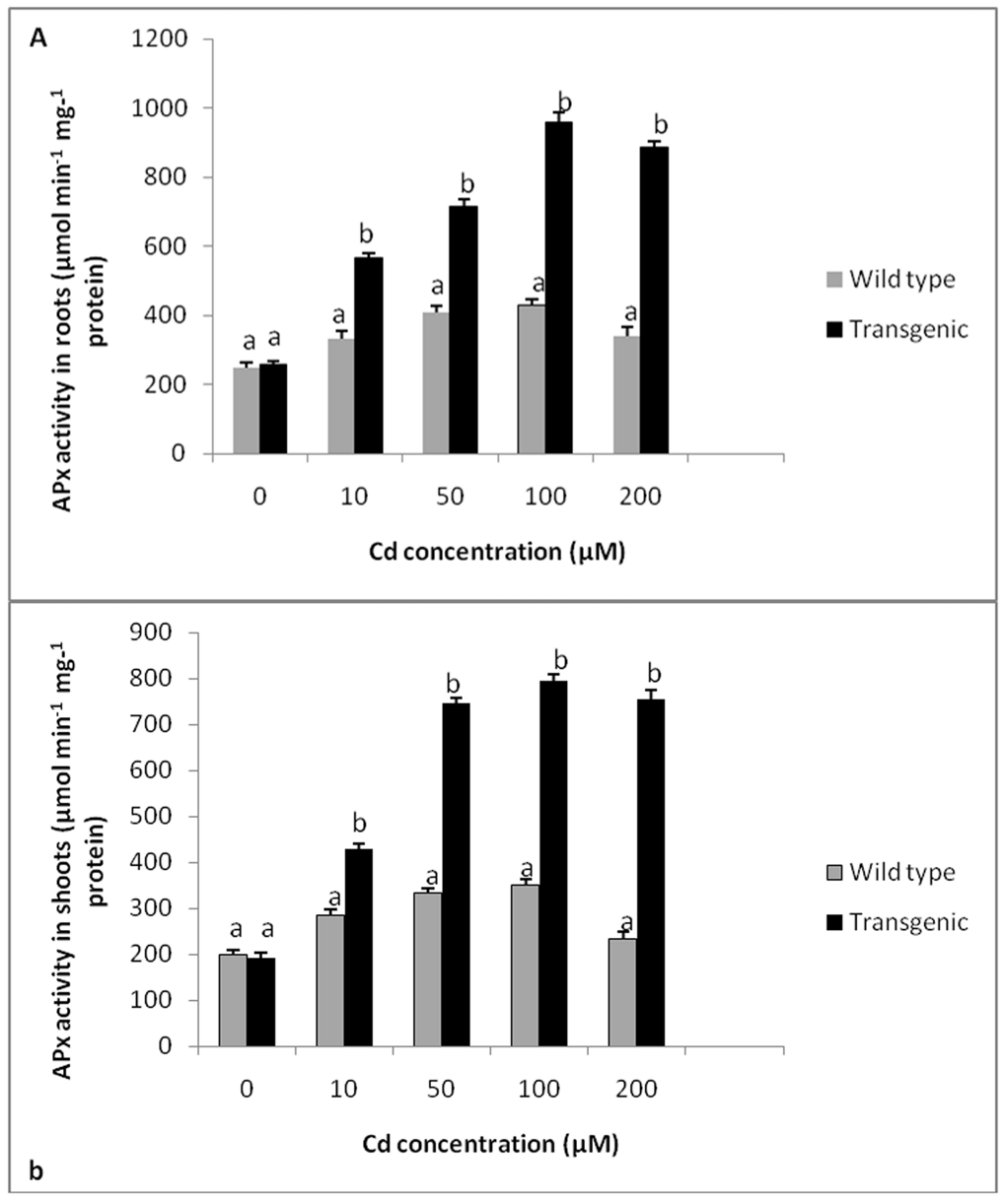
APX activity in transgenic and wild type plants exposed to different Cd concentrations. **A** APX activity in roots of transgenic and wild type plants exposed to different Cd concentrations. **B** APX activity in shoots of transgenic and wild type plants exposed to different Cd concentrations. All values are means of three replicates. Different letters indicate significantly different values at p≤0.05.

The levels of APX in roots and shoots of untreated wild type and transgenic tobacco plants expressing *TvGST* gene were found to be similar. With increasing concentrations of Cd, levels of APX increased in both wild type and transgenic plants. At all Cd concentrations, APX activity in roots of transgenic plants was significantly higher compared to wild type plants, with maximum activity observed at 100 µM Cd, which is about 2.2 times more compared to wild type plants. Roots of transgenic plants exhibited 3.1 times higher APX activity at 200 µM Cd concentration as compared to wild type plants (Figure 6A). A similar trend was observed for APX activity in shoots of wild type and transgenic plants, with transgenic plants showing significantly enhanced levels of APX activity in comparison with wild type plants (Figure 6B).

**Figure 7:**
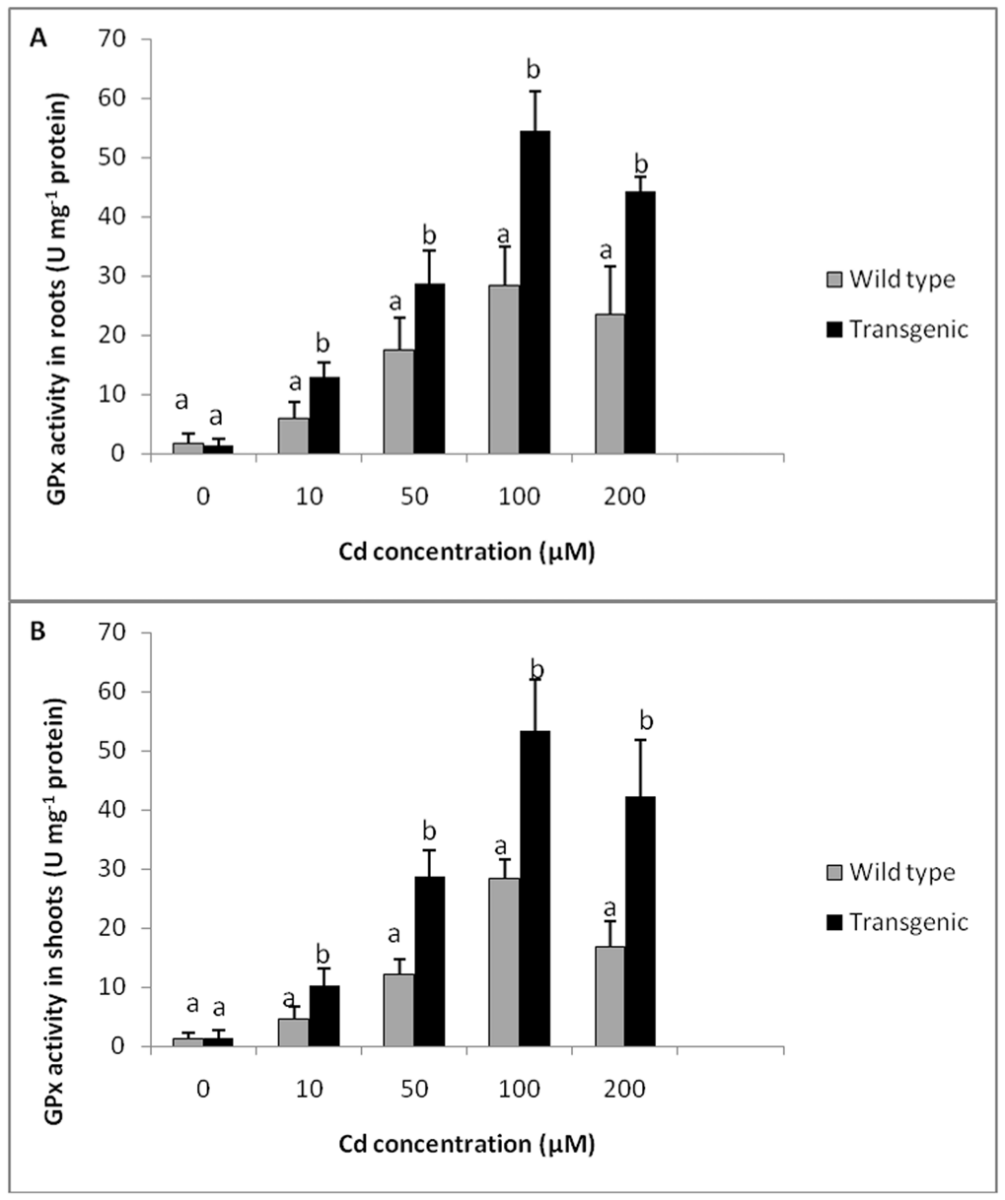
GPX activity in transgenic and wild type plants exposed to different Cd concentrations. **A** GPX activity in roots of transgenic and wild type plants exposed to different Cd concentrations. **B** GPX activity in shoots of transgenic and wild type plants exposed to different Cd concentrations. All values are means of three replicates. Different letters indicate significantly different values at p≤0.05.

Guiacol peroxidase activity in roots and shoots of wild type and transgenic plants also followed a similar trend. In the absence of Cd treatment, levels of GPX in roots and shoots of wild type plants and transgenic plants were similar. Roots and shoots of both transgenic and wild type plants showed an enhancement in GPX activity with increase in concentration of Cd. The levels of GPX in roots and shoots of transgenic plants were significantly higher compared to wild type plants at all Cd concentrations tested. Transgenic plants showed 1.9 times higher GPX activity in roots (Figure 7A), while shoots showed a maximum of 1.8 times more GPX activity as compared to wild type plants when exposed to 100 µM Cd (Figure 7B). The activity of GPX exposed to 200 µM Cd was found to be 2.3 times higher in roots and 3.4 times higher in shoots as compared to wild type plants. In general, transgenic plants exposed to different Cd concentrations showed enhanced levels of GPX compared to control plants.

**Figure 8:**
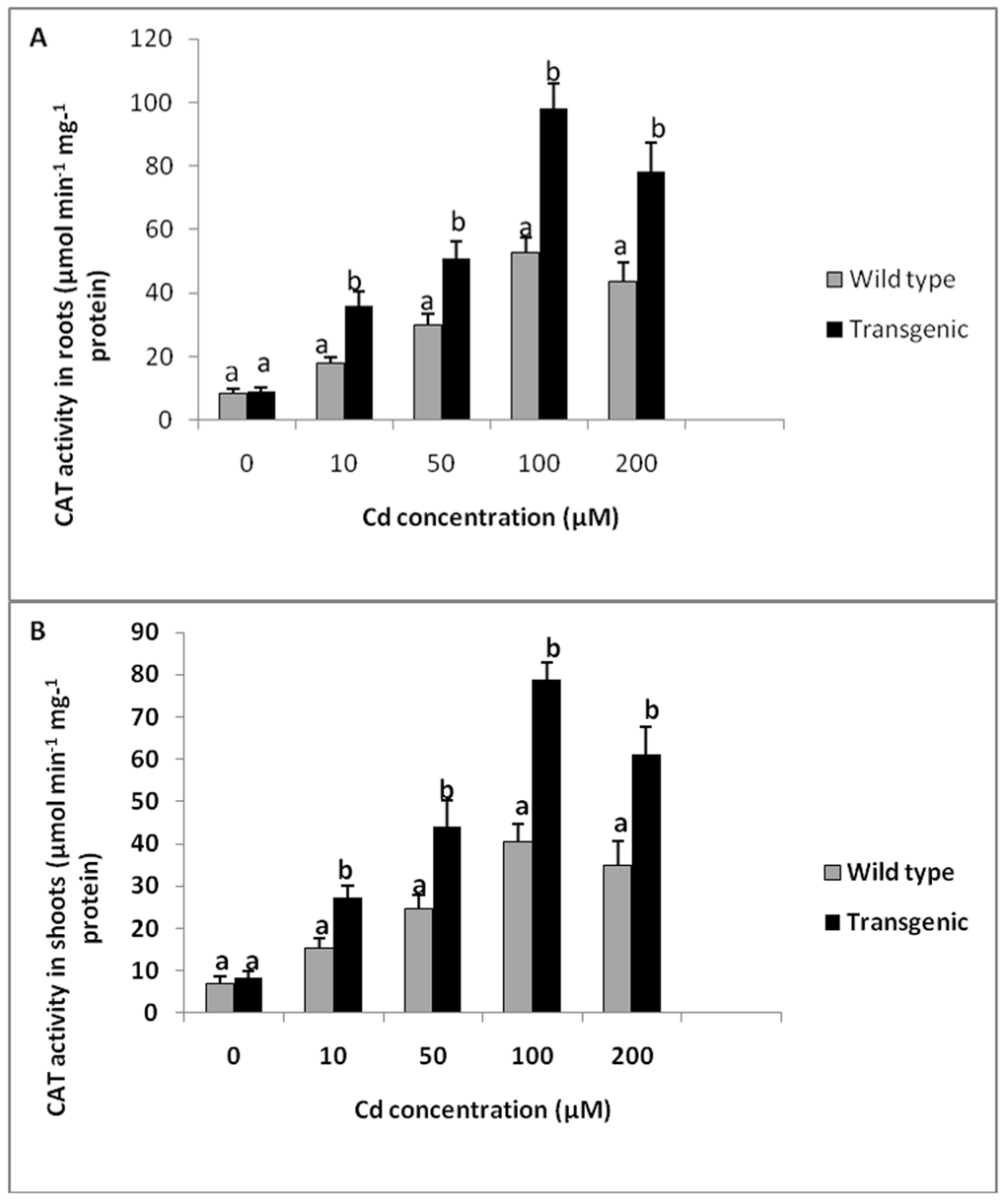
CAT activity in of transgenic and wild type plants exposed to different Cd concentrations. **A** CAT activity in roots of transgenic and wild type plants exposed to different Cd concentrations. **B** CAT activity in shoots of transgenic and wild type plants exposed to different Cd concentrations. All values are means of three replicates. Different letters indicate significantly different values at p≤0.05.

Catalase (CAT) activity in roots and shoots of wild type and transgenic tobacco plants without Cd treatment was found to be similar (Figure 8). With increase in Cd concentration, there was an increase in CAT activity in both transgenic and wild type plants. Catalase activity was significantly higher in transgenic plants as compared to control plants at all concentrations of Cd tested. Maximum CAT activity was seen in transgenic plants exposed to 100 µM Cd. Transgenic plants showed 1.8 times higher CAT activity in roots and 1.7 times higher activity in shoots at 100 µM Cd compared to wild type plants. CAT activity was 2.2 times higher in roots and 2.8 times higher in shoots of transgenic plants as compared to wild type plants exposed to 200 µM Cd (Figure 8A, B).

### Thiobarbituric acid reactive substances (TBARS) content

**Figure 9:**
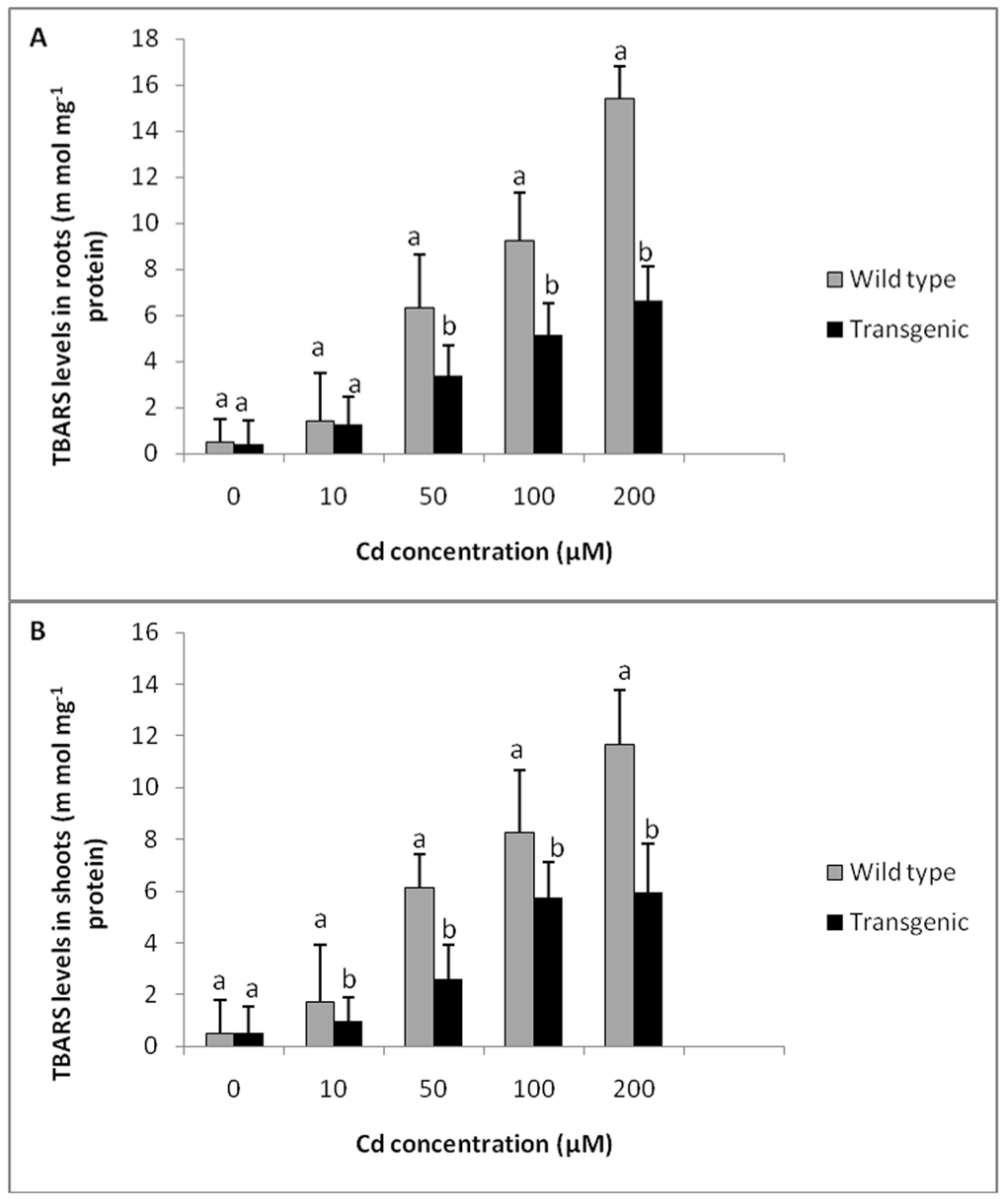
Effect of Cd treatment on TBARS concentration in transgenic and wild type plants. **A** Effect of Cd treatment on TBARS concentration in roots of transgenic and wild type plants exposed to different concentrations of Cd. **B** TBARS concentration in shoots of transgenic and wild type plants exposed to different Cd concentrations. All values are means of three replicates. Different letters indicate significantly different values at p≤0.05.

The levels of lipid peroxidation in terms of TBARS in roots and shoots of wild type and transgenic plants were similar without any Cd treatment (Figure 9). When plants were exposed to 10 µM Cd, levels of lipid peroxidation in roots of wild type plants and transgenic plants were comparable. Levels of TBARS increased significantly with exposure to increasing Cd concentrations in both wild type and transgenic plants. However, the levels of TBARS were found to be significantly lower in transgenic plants as compared to wild type plants exposed to 50, 100 and 200 µM of Cd. Roots and shoots of wild type plants showed 2.2 and 2.4 times higher TBARS content respectively at 200 µM Cd as compared to transgenic plants, indicating lower lipid peroxidation in transgenic plants compared to wild type plants (Figure 9 A, B).

### Cd accumulation

**Figure 10:**
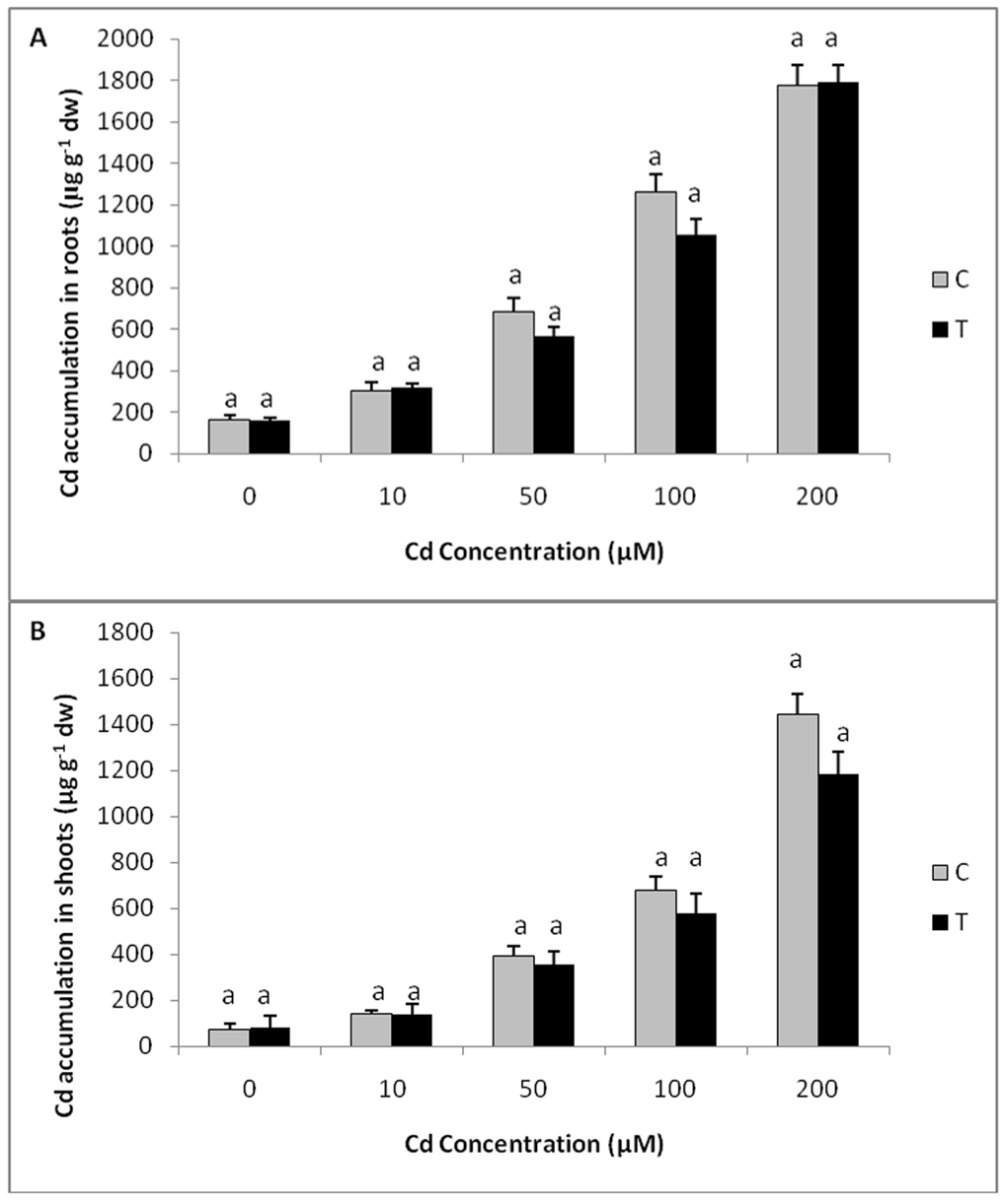
Cd accumulation in transgenic and control plants exposed to different concentrations of Cd. **A** Cd accumulation in roots of transgenic (T_0_) and control plants exposed to different concentrations of Cd. **B** Cd accumulation in shoots of transgenic and wild type plants exposed to different Cd concentrations. All values are means of three replicates. Different letters indicate significantly different values at p≤0.05.

**Figure 11:**
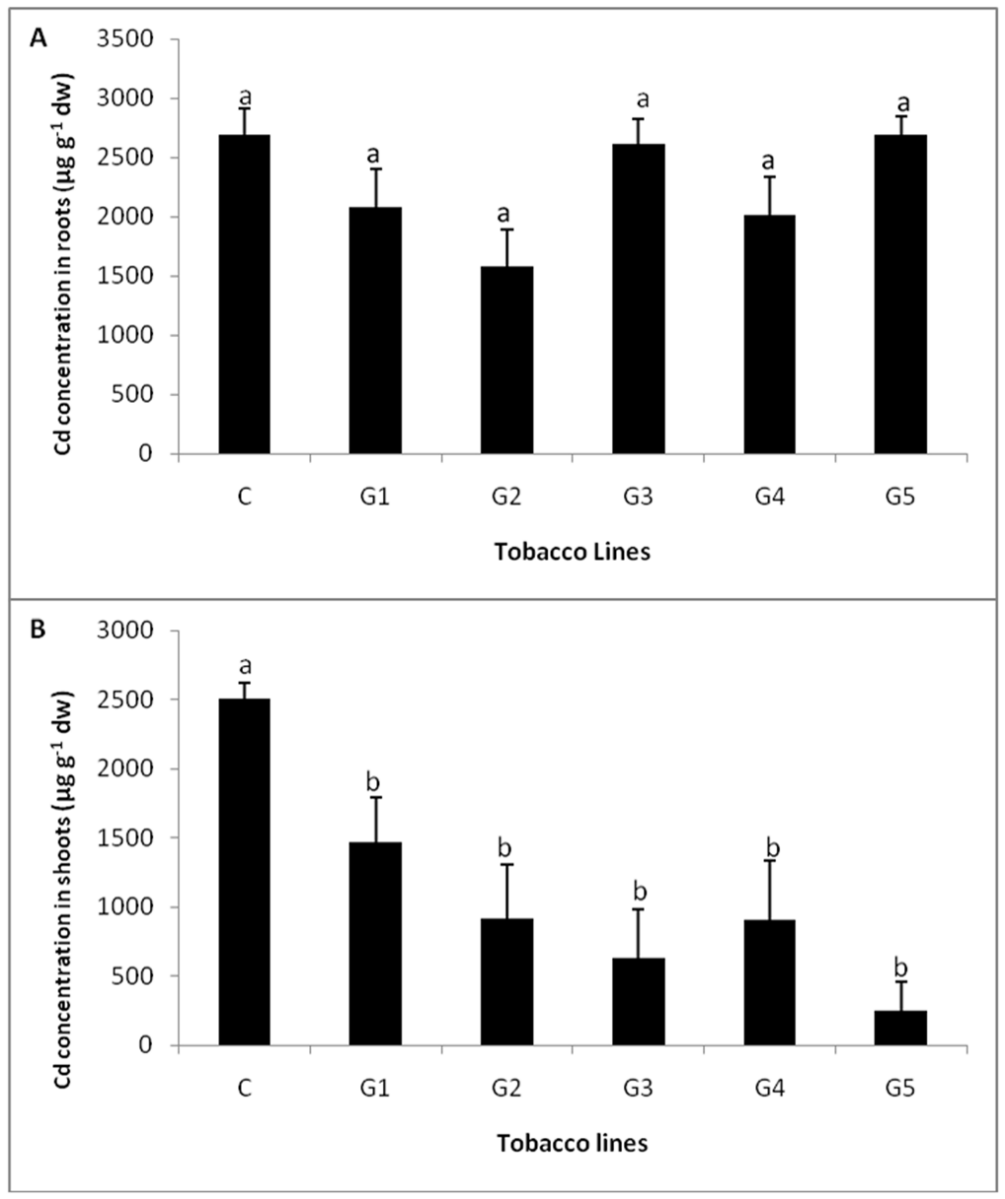
Cd accumulation in T_1_ transgenic and control plants exposed to different concentrations of Cd. **A** Cd accumulation in roots of five independent T_1_ transgenic and control plants exposed to 200 µM concentrations of Cd. **B** Cd accumulation in shoots of five independent T_1_ transgenic and control plants exposed to 200 µM concentrations of Cd. All values are means of three replicates. Different letters indicate significantly different values at p≤0.05.

When the levels of Cd in shoots and roots of T_0_ transgenic plants exposed to different concentrations of Cd were estimated, although there was an increase in Cd accumulation in shoot and roots with an increase in exposure levels of Cd, no significant difference in Cd accumulation was observed between control and transgenic plants at all the treatments (Figure 10 A, B). When Cd concentration was estimated in five independent T_1_ transgenic and wild-type plants, lower levels of Cd accumulation were observed in shoots of all the transgenic plants as compared to control plants while no significant difference in the levels of Cd accumulation was observed in roots of T_1_ transgenic and wild-type plants exposed to 200 µM Cd. (Figure 11 A, B).

## Discussion

Glutathione transferases are known to protect plants against oxidative stress induced by biotic and abiotic agents, by detoxifying endogenous cytotoxic compounds, which accumulate during oxidative stress. Since our earlier work with *Trichoderma virens* genes have shown good expression in plants [23], we chose a unique GST gene of *Trichoderma virens* for incorporation into tobacco for developing Cd tolerance. In the present study, transgenic tobacco plants expressing a *TvGST* gene were found to be tolerant to Cd, a toxic heavy metal which often contaminates agricultural fields. Transgenic plants showed better growth, enhanced tolerance, lower levels of lipid peroxidation and enhanced levels of antioxidant enzymes as compared to wild type plants when grown in the presence of Cd. Transgenic tobacco plants could grow at 100 µM of Cd without significant growth retardation, while growth of wild type plants was severely inhibited. Transgenic plants survived better than control even at 200 µM of Cd, although growth was marginally affected. The present study has shown that transgenic tobacco plants with *TvGST* gene are better equipped to combat Cd induced stress and could grow in the presence of toxic levels of Cd compared to wild type plants.

Plant cell membranes are the primary sites of metal injury, which is attributed to lipid peroxidation induced by ROS such as O_2_, OH or ^1^O_2_ or by action of lipooxygenases [25]. Heavy metals such as Cd are known to induce ROS through a series of redox reactions leading to lipid peroxidation and oxidative stress. The levels of TBARS, an indicator of lipid peroxidation, were significantly lower in transgenic plants expressing a *TvGST* gene as compared to control when exposed to Cd. Since, Cd-induced lipid peroxidation was lower in transgenic plants, they could grow better in the presence of Cd compared to control plants. Hence, the present work has shown that expression of *TvGST* gene could impart Cd tolerance in tobacco. Earlier workers have shown that transgenic plants which overexpressed plant GST/ peroxidase showed enhanced tolerance to different abiotic stresses [26]. For example, when *Suadea salsa* GST was expressed in *Arabidopsis*, the plants showed tolerance to salt stress and had lower levels of lipid peroxidation [27].

Plants have an efficient antioxidant system to maintain the correct balance between overproduction of ROS and their scavenging to keep them at the required levels. Enzymes such as SOD, CAT, APX, GPX and GSTs, catalyze the scavenging of ROS and combat oxidative stress induced by different agents including heavy metals. Transgenic tobacco plants with a *TvGST* gene (present study) showed higher levels (about 7 fold) of GST in the absence of any treatment as well as when exposed to Cd, compared to wild type plants. Some GSTs also have secondary activities as glutathione peroxidase and can protect the cells/organisms from oxidative damage induced by metals such as Cd. GSTs can eliminate membrane lipid peroxides as well as products of oxidative DNA degradation by conjugating them with GSH [4]. Transgenic tobacco plants expressing a fungal GST gene (present study), besides having enhanced levels of GST, also showed higher levels of SOD, APX, GPX and CAT compared to wild plants when exposed to Cd, indicating that these transgenic plants expressing *TvGST* are better equipped with antioxidant defense system to combat Cd induced oxidative stress. The present observation that the levels of antioxidant enzymes such as APX, GPX, CAT, SOD and GST showed an enhancement when exposed to Cd in transgenics is in accordance with the earlier studies [28]–[30]. The co-regulation of GST, POX, CAT and SOD expression could be part of a system for regulating the balance between the detrimental and beneficial roles of H_2_O_2_ in plant cells [31]. The enhancement in the levels of SOD, APX, GPX, CAT and GST activity in GST transgenics as demonstrated in the present study imparted enhanced tolerance to Cd. However, further studies need to be carried out to understand how the presence of *TvGST* gene enhances expression of other related antioxidant enzymes when exposed to Cd.

The present study has shown that the levels of Cd accumulation in transgenic plants and wild type plants were comparable with control when exposed to varying concentrations of Cd. Transgenic tobacco plants expressing a fungal GST although were found to be more tolerant to Cd, the levels of Cd accumulation were comparable/ lower than control. Hence, *TvGST* gene expression in transgenic tobacco, as shown in the present study, imparted Cd tolerance to plants, but did not enhance its accumulation. There are earlier reports on enhancement in Cd tolerance in transgenic plants by incorporation of different genes such as yeast metallothionien gene *CUP*1 [32], *Arabidopsis* Ca^2+^/H^+^ antiporter CAX1 [20], *B. juncea* BjCdR15 [21], *Arabidopsis* phytochelatin synthase [22] and soybean O-acetylserine (thiol) lyase-encoding gene GmOASTL4 [33]. However, in those studies, most of the Cd tolerant transgenic plants also showed enhanced Cd accumulation compared to wild type plants. Cadmium accumulation will be a useful trait for developing plants for phytoremediation [34]–[36], but is not a desirable trait when the objective is to develop crop plants tolerant to Cd, as enhanced accumulation of Cd is not desired.

In the changing scenario of increasing heavy metal contamination in the environment due to altered agricultural practices and increasing anthropogenic activities, it is necessary to develop crop plants which can tolerate heavy metals without accumulating them in edible parts. In the present study, we have used tobacco as a model plant to develop Cd tolerance by heterologous expression of a fungal GST gene. Tobacco plants, thus developed could tolerate Cd without any enhancement in Cd accumulation. This can be extended for development of crop plants tolerant to Cd. It will also help in limiting Cd bioavailability in plant based foods even when the soil is contaminated with Cd.

## Materials and Methods

### Cloning and sequence analysis of the *Trichoderma virens* glutathione transferase

The partial sequence of *Trichoderma virens* (strain IMI 304061) glutathione transferase cDNA (GenBank Acc. EH628505) was obtained from our ESTs database (GenBank Acc. Nos. EH628893 to EH628394); this was the unique GST gene identified among nearly 800 cDNA clones sequenced in our laboratory (Mukherjee PK, unpublished). We amplified, cloned and sequenced the full-length gene by genome walking using the primers 5′-CGA TCC GTC GGC CCA CTT GTC AGT G-3′ (BD Universal Genome Walking Kit). The introns were identified by alignment with the cDNA sequence. The full length cDNA sequence was submitted to GenBank (Acc. GU014698). The homology of the translated sequence was searched using the BLASTP algorithm (http://ncbi.nlm.nih.gov/BLAST) and phylogenetic analysis done using CLUSTALW software (http://align.genome.jp/).

### Construction of plant expression vector

*TvGST* gene was cloned in the binary vector pCAMBIA 1301 (CAMBIA, Australia; Supplementary Information, SI). Resulting plasmid (pGST) harboured *TvGST*, *uid*A (reporter) and *hph* (plant selectable marker) genes (Figure S7). Plasmid pGST was introduced into *Agrobacterium tumefaciens* strain EHA105 via electroporation using electroporator 2510 (Eppendorf, Germany).

### Transformation of tobacco

Leaf discs of tobacco (*Nicotiana tabacum* L.) cv. Havana 425, were transformed with *A. tumefaciens* harbouring pGST using the method described by Horsch et al [37]. The explants after cocultivation for 2 days were transferred to MS medium supplemented with 2 mgL^−1^ benzyladenine, 0.1 mgL^−1^ indole acetic acid, 25 mgL^−1^ hygromycin and 500 mgL^−1^ cefotaxime. The developed shoots were subcultured on MS medium to develop roots. Histochemical assay for the reporter gene (*uid*A) product was done according to the procedure by Jefferson [38] at the end of 48 h of coculture for transient expression and 5–6 weeks after transformation for stable expression.

### Molecular analyses of the transgenic lines

Total genomic DNA from the leaves of putatively transformed and control tobacco plants was isolated [39] and subjected to PCR amplification using specific primers for *TvGST* gene (Table S1). Integration of *TvGST* was further confirmed by Southern blot hybridization. In brief, thirty µg genomic DNA of putatively transgenic and control plants were digested with *Hind*III enzyme and transferred to a positively charged nylon membrane (Boehringer Mannheim, Germany). Probe was prepared using PCR product of *TvGST* gene using DIG labeling kit (Roche Biochemicals, Germany). Prehybridization, hybridization, washing and detection were carried out using chemiluminescence detection kit (Roche, Germany) according to the manufacturer's instructions.

Transcription of *TvGST* gene was confirmed by conducting reverse transcription PCR using Affinity Script Multi Temperature cDNA synthesis Kit (Stratagene, U.S.A) using gene specific primers (Table S1).

### Analysis of T_1_ plants

To investigate the inheritance of hygromycin resistant trait, six independent primary transgenic lines (T_0_) were selected and self pollinated by bagging. Seeds were harvested and germinated on MS medium for one month. Almost 95% seeds were found to be viable. Germinated seedlings were then transferred to MS medium containing hygromycin B (25 mg l^−1^). After 4 weeks, number of hygromycin resistant seedling with well developed roots and leaves which were clearly distinguished from the sensitive seedlings with pale green cotyledons and less developed root system were counted and chi-square test was done to determine the segregation pattern of the transgene. PCR confirmed T_1_ generation plants from each of the six T_0_ lines (highly expressing GST gene) were assayed for the expression of GST gene. Leaf tissue of PCR confirmed T_1_ plants were taken and enzyme assay for GST gene was conducted.

### GST extraction and assay

Leaf tissues (0.2 g) from fifteen day old transgenic and control plants grown in MS medium were homogenized in 100 mM phosphate buffer, pH 7.0 containing 0.05 mM DTE, 1 mM EDTA and 3.5% (w/v) PVPP. Slurry was centrifuged at 10000 g for 3 minutes, pellet discarded and the supernatant used as the crude enzyme extract for the GST assay. Glutathione transferase activity was assayed spectrophotometrically at 340 nm by measuring the rate of 1-chloro-2, 4-dinitrobenzene conjugation with reduced glutathione as a function of time according to the method by Habig and Jacoby [40]. The assay mixture contained 0.1 ml 30 mM glutathione, 0.1 ml (approximately 2–3 mg protein/ml) plant extract, 0.1 ml 30 mM CDNB and 2.7 ml 100 mM phosphate buffer, pH 6.5. The enzyme activity was expressed as µmol^−1^min^−1^ and the specific activity as µmolmin^−1^mg^−1^ protein. All results were average of three replicates. Protein contents were determined by the method of Lowry et al [41] using bovine serum albumin as a protein standard.

### Cadmium tolerance and assay

For studying tolerance to Cd, T_0_ of one transgenic tobacco line (G11) showing highest expression of GST and five T_1_ confirmed transgenic lines were selected and used for the experiments. Transgenic plants along with wild type plants having similar biomass (0.2 gm each) in triplicates (multiplied by micropropagation for getting replicates of same genotype) were transferred to Hoagland's medium [42] for 10 days for acclimatization. Subsequently, T_0_ and T_1_ plants were exposed to different concentrations of cadmium i.e., 0, 5, 10, 50, 100, 200 and 400 µM for a period of 15 days. Observations were recorded for the growth of the plants exposed to Cd and at the end of 15 days, plants were harvested, fresh weight recorded and antioxidant enzymes estimated. Antioxidant estimation was done only for T_0_ plants exposed to Cd. Further, roots and shoots were separated, washed with distilled water and oven dried at 70°C for one week. Dried plant tissues were ground, weighed and digested in an acid mixture of HNO_3_: HClO_4_ (5∶1, v/v) at 80°C and Cd content was estimated by GBC 932 B+ Atomic Absorption Spectrophotometer (GBC Scientific Equipment, Melbourne, Australia) using air-acetylene flame.

### Determination of antioxidant enzymes

Roots and shoots of the control and treated plants (T_0_) harvested after 15 days were homogenized in 100 mM Na-Phosphate buffer, pH 7. The homogenate was centrifuged at 12,000 g for 10 min at 4°C. All steps in the preparation of enzyme extract were carried out at 0–4°C. This supernatant was used to measure the activities of SOD, APX, GPX and CAT enzymes at 25°C using a spectrophotometer.

### Glutathione transferase (EC 2.5.1.18)

Roots and shoots of the control and transgenic plants exposed to Cd were harvested after 15 days and GST activity was assayed [40].

### Guaiacol Peroxidase (EC 1.11.1.7)

Guaiacol peroxidase was measured in the roots and shoots of Cd treated transgenic and wild type plants using the method of Curtis [43], modified by Kato and Shimizu [44]. Activity was calculated using the extinction coefficient as 26.6 mM^−1^ cm^−1^ at 470 nm for oxidized tetraguiacol polymer. One unit of peroxidase activity was defined as the consumption of 1 µmol of H_2_O_2_ min^−1^ g^−1^ f w.

### Ascorbate Peroxidase (EC 1.11.1.11)

Ascorbate peroxidase (APX) activity in the roots and shoots of transgenic and wild type tobacco plants treated with cadmium was determined by the method of Nakano and Asada [45], which estimates the rate of ascorbate oxidation at 290 nm (extinction coefficient: 2.8 mM^−1^ cm^−1^). The enzyme activity was expressed in terms of µmol of ascorbate oxidized min^−1^ g^−1^ f w.

### Catalase (EC 1.11.1.6)

Catalase (CAT) was estimated in transgenic and control plants exposed to Cd following the method of Aebi [46]. The activity was estimated by monitoring the decrease in absorbance due to H_2_O_2_ reduction (extinction coefficient: 39.4 M^−1^cm^−1^) at 240 nm. The activity was expressed in terms of µmol of H_2_O_2_ reduced min^−1^ g^−1^ f w at 25±2°C.

### Lipid peroxidation

The level of lipid peroxidation, in terms of thiobarbituric acid reactive substances (TBARS) content in the plant samples (roots and leaves of transgenic and control) was estimated by thiobarbituric acid (TBA) reaction [47].

### Statistical analysis

All the experiments were carried out in triplicates and repeated at least twice using completely randomized design (CRD). One way Analysis of variance (ANOVA) was used to confirm variability of data and validity of results. To determine the levels of significance between transgenic and control lines, Least Significance Difference (LSD) was calculated using software IRRISAT.

## Supporting Information

Figure S1 Comparison of *TvGST* with other Ascomycetes fungi. Ptri- *Pyrenophora tritici repentis* Cimi- *Coccidioides immitis* Pmar-*Penicillium marneffei* Acia- *Aspergillus clavatus* Anig- *Aspergillus niger* Afum-*Aspergillus fumigates* Nfis- *Neosartorya fischeri* Anid-*Aspergillus nidulans* Aoty- *Aspergillus oryzae* Ater *Aspergillus terreus* Bfuc-*Botyyotinia fuckeliana* Sscl- *Sclerotinia sclerotiorum* Cglo- *Colletotrichum gloeosporioides* Pans- P*odospora anserine* Gzea-*Gibberella zeae* Hvir-*Hypocera virens* Mgri-*Magnaporthe grisea* Ncra- *Neurospora crassa.*
(TIF)

Figure S2 Histochemical GUS assay, blue coloration in the explants shows the expression of *uidA* gene. a) Transient Gus assay b) Gus assay in regenerating leaf disc c) Stable gus assay in leaf.
(TIF)

Figure S3 PCR amplification for GST gene from genomic DNA of putative transgenic plants. a) 1 pCAMBIA GST-1301 plasmid b) 2 DNA from non transgenic tobacco plant c) 3-15 DNA from putative transgenic tobacco plants.
(TIF)

Figure S4 Effect of Cd treatment on growth of wild type and transgenic plants exposed to different concentrations of Cd.
(TIF)

Figure S5 Effect of Cd treatment on growth of wild type and five independent T_1_ transgenic plants exposed to different concentrations of Cd.
(TIF)

Figure S6 Effect of Cd treatment on growth of wild type and T_1_ transgenic plants exposed to 100 µM (a) and 200 µM (b) concentrations of Cd.
(TIF)

Figure S7 T-DNA region of pGST plasmid harbouring *TvGST* gene. T-DNA region of pCAMBIA 1301 vector harbouring GST gene under 35S-35-S CaMV promoter, AMV translational enhancer element and nos terminator. LB and RB are the left and right T-DNA borders. **Construction of plant expression vector.** Sites for the restriction enzymes *Nco*I and *Xba*I were incorporated in the forward and reverse primers, respectively and the amplified product was cloned into pTZ57R/T (Fermentas) cloning vector to get the desired restriction sites for subsequent cloning purpose. Gene sequence was confirmed by DNA sequencing (MWG, Bangalore) and subcloned into pBI525 vector under the control of double 35S cauliflower mosaic virus promoter (CaMV), alfalfa mosaic virus (AMV) 5′ untranslated leader sequence as translational enhancer and nopaline synthase terminator (nos) as terminator. The resulting vector was digested with *EcoR*I and *Hind*III restriction enzymes, which excises 2 Kb cloning cassette containing 5′-35S-35S-CaMV-AMV-*GST*-nosT-3′and finally cloned into binary plant expression vector pCAMBIA1301 (CAMBIA, Australia) harboring *hph* as plant selectable marker and *uidA* as reporter gene. Resulting plasmid pGST, harboring the *T. virens* GST ORF (*TvGST*) under the 35S-35S-CaMV promoter was introduced into *Agrobacterium tumefaciens* EHA 105 strain using an electroporator (Eppendorf, Germany).
(TIF)

Table S1 Primer sequences used.
(DOC)

Table S2 Study of inheritance in T_1_ tobacco. At p<0.05 and n=1, χ^2^ value is 3.84, hence, χ^2^ value from all lines were found to be significant. s were found to be significant.
(DOC)
