# Supplementary figures and images for: Glutathione Transferase from Trichoderma virens Enhances Cadmium Tolerance without Enhancing Its Accumulation in Transgenic Nicotiana tabacum
Source: PLoS One. 2011 Jan 21;6(1):e16360. doi: 10.1371/journal.pone.0016360 (PMC3024989; doi:10.1371/journal.pone.0016360)

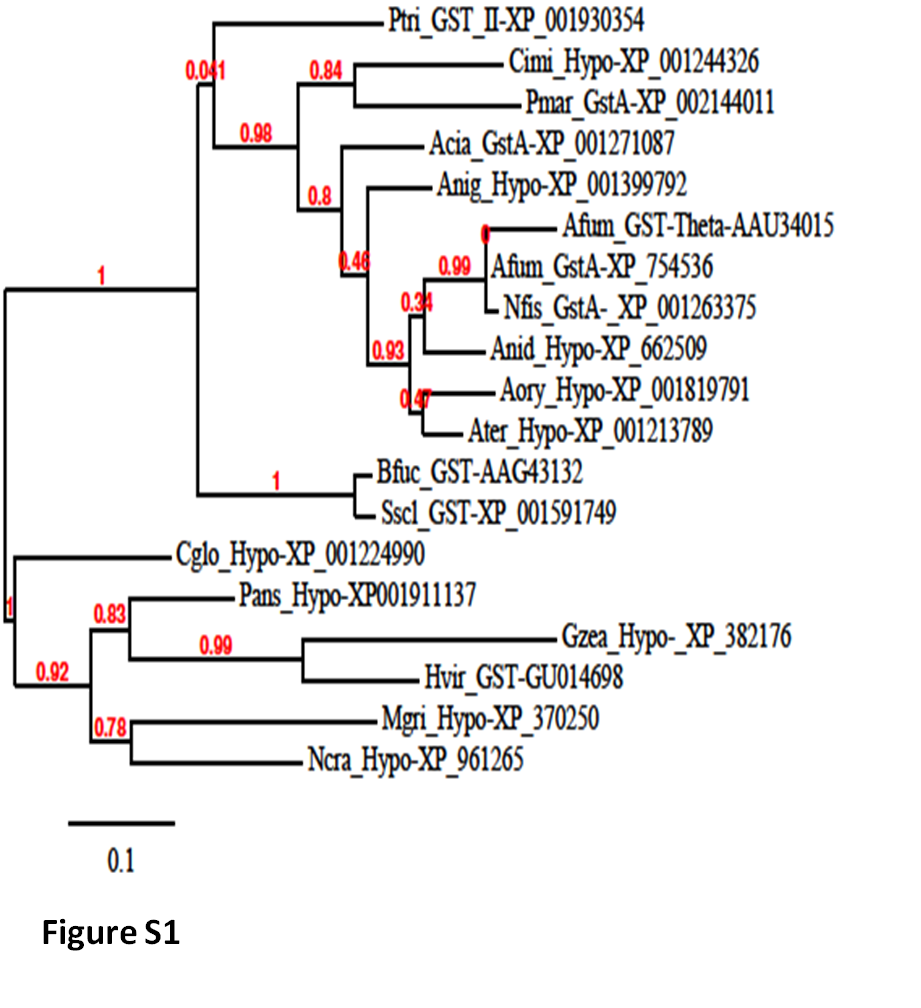

Supplement: Figure S1 — Comparison of TvGST with other Ascomycetes fungi. Ptri- Pyrenophora tritici repentis Cimi- Coccidioides immitis Pmar-Penicillium marneffei Acia- Aspergillus clavatus Anig- Aspergillus niger Afum-Aspergillus fumigates Nfis- Neosartorya fischeri Anid-Aspergillus nidulans Aoty- Aspergillus oryzae Ater Aspergillus terreus Bfuc-Botyyotinia fuckeliana Sscl- Sclerotinia sclerotiorum Cglo- Colletotrichum gloeosporioides Pans- Podospora anserine Gzea-Gibberella zeae Hvir-Hypocera virens Mgri-Magnaporthe grisea Ncra- Neurospora crassa. (TIF) [file pone.0016360.s001.tif]

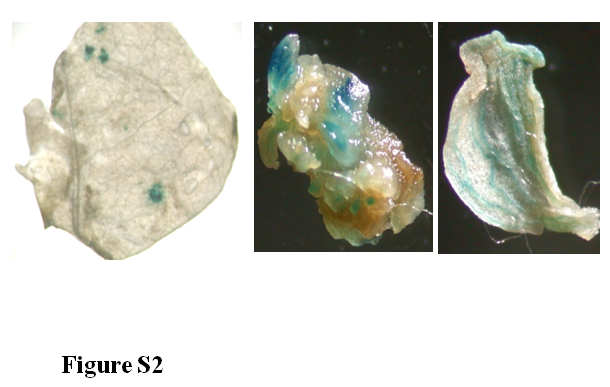

Supplement: Figure S2 — Histochemical GUS assay, blue coloration in the explants shows the expression of uidA gene. a) Transient Gus assay b) Gus assay in regenerating leaf disc c) Stable gus assay in leaf. (TIF) [file pone.0016360.s002.tif]

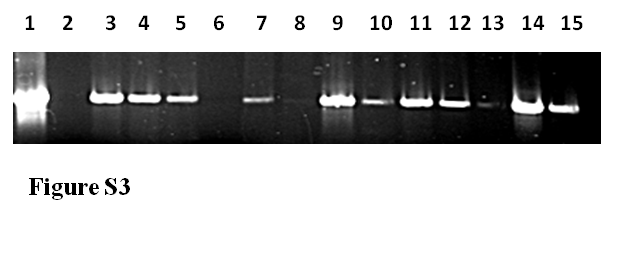

Supplement: Figure S3 — PCR amplification for GST gene from genomic DNA of putative transgenic plants. a) 1 pCAMBIA GST-1301 plasmid b) 2 DNA from non transgenic tobacco plant c) 3-15 DNA from putative transgenic tobacco plants. (TIF) [file pone.0016360.s003.tif]

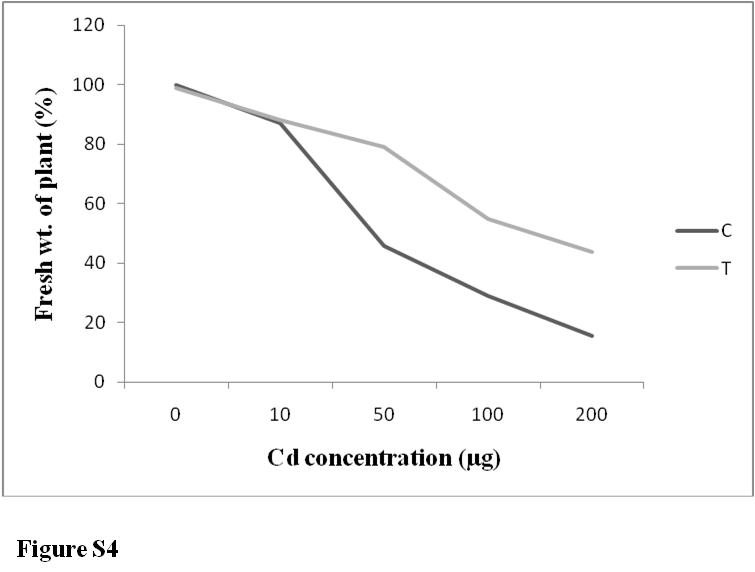

Supplement: Figure S4 — Effect of Cd treatment on growth of wild type and transgenic plants exposed to different concentrations of Cd. (TIF) [file pone.0016360.s004.tif]

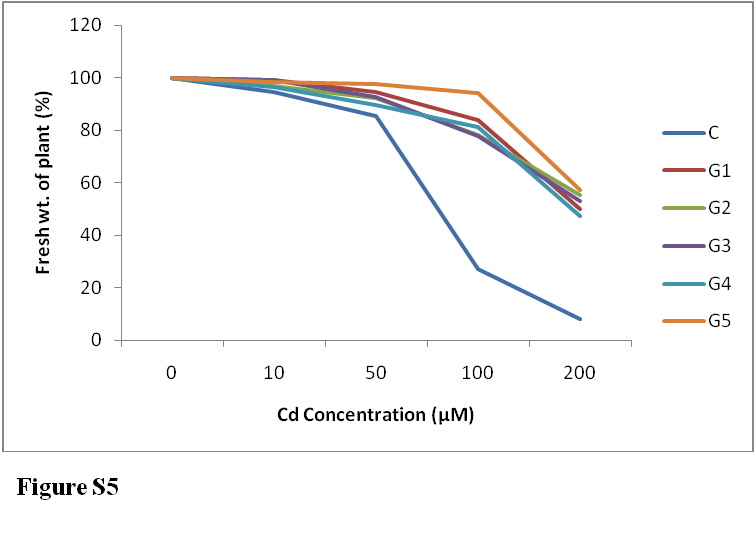

Supplement: Figure S5 — Effect of Cd treatment on growth of wild type and five independent T1 transgenic plants exposed to different concentrations of Cd. (TIF) [file pone.0016360.s005.tif]

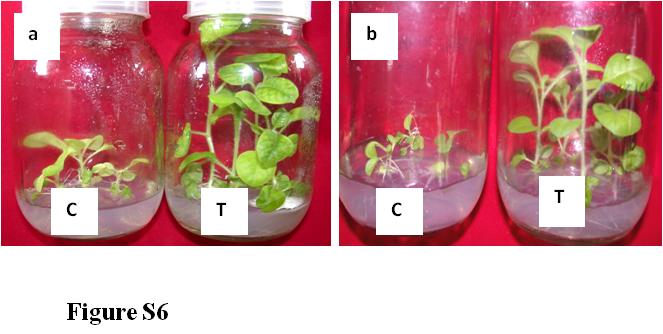

Supplement: Figure S6 — Effect of Cd treatment on growth of wild type and T1 transgenic plants exposed to 100 µM (a) and 200 µM (b) concentrations of Cd. (TIF) [file pone.0016360.s006.tif]

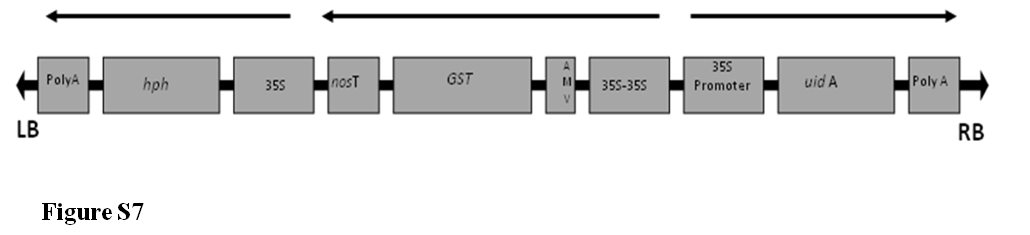

Supplement: Figure S7 — T-DNA region of pGST plasmid harbouring TvGST gene. T-DNA region of pCAMBIA 1301 vector harbouring GST gene under 35S-35-S CaMV promoter, AMV translational enhancer element and nos terminator. LB and RB are the left and right T-DNA borders. Construction of plant expression vector. Sites for the restriction enzymes NcoI and XbaI were incorporated in the forward and reverse primers, respectively and the amplified product was cloned into pTZ57R/T (Fermentas) cloning vector to get the desired restriction sites for subsequent cloning purpose. Gene sequence was confirmed by DNA sequencing (MWG, Bangalore) and subcloned into pBI525 vector under the control of double 35S cauliflower mosaic virus promoter (CaMV), alfalfa mosaic virus (AMV) 5′ untranslated leader sequence as translational enhancer and nopaline synthase terminator (nos) as terminator. The resulting vector was digested with EcoRI and HindIII restriction enzymes, which excises 2 Kb cloning cassette containing 5′-35S-35S-CaMV-AMV-GST-nosT-3′and finally cloned into binary plant expression vector pCAMBIA1301 (CAMBIA, Australia) harboring hph as plant selectable marker and uidA as reporter gene. Resulting plasmid pGST, harboring the T. virens GST ORF (TvGST) under the 35S-35S-CaMV promoter was introduced into Agrobacterium tumefaciens EHA 105 strain using an electroporator (Eppendorf, Germany). (TIF) [file pone.0016360.s007.tif]
