# Supplementary material for: Glutathione Transferase from Trichoderma virens Enhances Cadmium Tolerance without Enhancing Its Accumulation in Transgenic Nicotiana tabacum
Source: PLoS One. 2011 Jan 21;6(1):e16360. doi: 10.1371/journal.pone.0016360 (PMC3024989; doi:10.1371/journal.pone.0016360)
Supplement: Table S1 — Primer sequences used. (DOC) [file pone.0016360.s008.doc]

**Table S1** Primers used in PCR amplification

| **Primer** | **Expected size of product** | **Sequence** |
| --- | --- | --- |
| TvGSTF | 1008bp | 5’-CGG CAA TTG ACC AGC ACC ATG GGT ATC-3’ |
| TvGSTR |  | 5’-CTG GTA TCT AGA ATC ACT CAT TTC TTC-3’ |
| hphF | 450bp | 5’-GAG GGC GAA GAA TCT CGT GC-3’ |
| hphR |  | 5’- CAC TGA CGG TGT CGT CCA TC-3’ |
| uidAF | 1300bp | 5’-ATG GAT AAC AAT CCG AAC ATC AAA GA-3’ |
| uidAR |  | 5’-TTA TTA GCC CTA GTT GGT TTG TAC A-3’ |
