# Supplementary material for: Glutathione Transferase from Trichoderma virens Enhances Cadmium Tolerance without Enhancing Its Accumulation in Transgenic Nicotiana tabacum
Source: PLoS One. 2011 Jan 21;6(1):e16360. doi: 10.1371/journal.pone.0016360 (PMC3024989; doi:10.1371/journal.pone.0016360)
Supplement: Table S2 — Study of inheritance in T1 tobacco. At p<0.05 and n=1, χ2 value is 3.84, hence, χ2 value from all lines were found to be significant. s were found to be significant. (DOC) [file pone.0016360.s009.doc]

**Table S2**

**Segregation analysis of T_1_ generation**

T_0_ seeds were germinated and T_1_ seedlings were tested for segregation of *hph* gene. At 25 mg l^-1^, the green seedlings with well developed roots were classified as hygromycin resistant and they were easily distinguished from pale seedlings with poor roots, which were classified as hygromycin sensitive. Segregation analysis with the T_1_ generation showed a Mendelian ratio of 3:1 in all the tested lines (Table S2). At P<0.05, n=1, all values fit into the hypothesis that the transgene segregated in the Mendelian ratio of 3:1 and integration was at a single site. T_1_ hygromycin resistant transgenic plants showed the presence of *TvGST* gene on PCR amplification, confirming the presence of this transgene in T_1_ plants.

| Line | Resistant seedlings | Sensitive seedlings | X^2^ test  3:1 |
| --- | --- | --- | --- |
| Control | 0 | 150 | - |
| T1 | 112 | 38 | 0.008 |
| T2 | 120 | 30 | 2.0 |
| T3 | 105 | 45 | 2.0 |
| T4 | 114 | 36 | 0.08 |
| T5 | 122 | 28 | 3.2 |
| T6 | 118 | 32 | 1.07 |

At p<0.05 and n=1, χ^2^ value is 3.84, hence, χ^2^ value from all lines were found to be significant.
